# Impact of glucocorticoids on patients’ quality of life: a qualitative study assessing face validity and feasibility of the Steroid PRO in patients with inflammatory gastroenterology, respiratory and dermatology conditions

**DOI:** 10.1136/bmjopen-2024-089225

**Published:** 2025-02-05

**Authors:** Anne-Marie T Sweeney, Susan Bridgewater, Jen Orme, Sebastian E Sattui, Michelle Sharp, Pamela Richards, Christine A Silverthorne, Elizabeth Arthurs, Tom Creed, Genevieve Osborne, Giles Dunhill, Jill Dawson, Emma Dures, Shaney L Barratt, Richard P Ramonell, Timothy Patton, Susan M Goodman, Catherine L Hill, Sarah L Mackie, Mwidimi Ndosi, Joanna C Robson

**Affiliations:** 1 University Hospitals Bristol and Weston NHS Foundation Trust, Bristol, UK; 2 Center for Health and Clinical Research, University of the West of England, Bristol, UK; 3 University of Pittsburgh, Pittsburgh, Pennsylvania, USA; 4 Johns Hopkins University School of Medicine, Baltimore, Maryland, USA; 5 Gastroenterology Department, University Hospitals Bristol and Weston NHS Trust, Bristol, UK; 6 Dermatology Department, University Hospitals Bristol and Weston NHS Trust, Bristol, UK; 7 Nuffield Department of Population Health, University of Oxford, Oxford, UK; 8 University of the West of England, Bristol, UK; 9 North Bristol NHS Foundation Trust, Bristol, UK; 10 Division of Pulmonary Allergy Critical Care and Sleep Medicine, University of Pittsburgh, Pittsburgh, Pennsylvania, USA; 11 Department of Dermatology, University of Pittsburgh, Pittsburgh, Pennsylvania, USA; 12 Weill Cornell Medicine, New York City, New York, USA; 13 The University of Adelaide, Adelaide, South Australia, Australia; 14 Department of Rheumatology, The Queen Elizabeth Hospital, Woodville South, South Australia, Australia; 15 Leeds Biomedical Research Centre, Leeds Teaching Hospitals NHS Trust, Leeds, UK; 16 Leeds Institute of Rheumatic and Musculoskeletal Medicine, University of Leeds, Leeds, UK

**Keywords:** Asthma, Eczema, Gastroenterology, Dermatology, QUALITATIVE RESEARCH

## Abstract

**Abstract:**

**Objectives:**

The Steroid PRO is a treatment-specific patient-reported outcome questionnaire which measures the impact of glucocorticoids on health-related quality of life. It has 15 items grouped into 4 domains (Social impact, Impact on Appearance, Psychological Impact and Treatment Concerns). Initially developed and validated in rheumatic diseases, the Steroid PRO demonstrates potential for broader application in patients with other inflammatory conditions. The objective of this study was to assess face validity, content validity and feasibility of the Steroid PRO in (1) patients treated with glucocorticoids for inflammatory respiratory, dermatological and gastroenterological conditions and (2) clinicians working within these specialties in the UK and USA.

**Design:**

Qualitative study with semistructured cognitive interview methods.

**Setting:**

Online or face-to-face interviews with participants from seven departments across three secondary care hospitals in the UK and USA.

**Participants:**

Inclusion criteria: (1) Adult patients with inflammatory respiratory, gastroenterological and dermatological conditions treated with glucocorticoids and (2) healthcare professionals (HCPs) working in respiratory, dermatology and gastroenterology departments in the UK and USA.

**Results:**

Purposive sampling to ensure a range of patient and HCP participants. A total of 42 patient participants were recruited, from respiratory/pulmonology (n=14, 33.3%), dermatology (n=13, 31.0%) and gastroenterology (n=15, 35.8%) medical departments; 32 in the UK and 10 from the USA. Mean age 48.2 years (range 22–71) and 19 (45.2%) were female. Patient participants had a range of inflammatory lung, skin and bowel conditions, with a spectrum of demographics and patterns of glucocorticoid use. 14 HCPs participated from the UK (9) and USA (5). Face validity: 97% (30/31) patients and 100% (14/14) HCPs reported the Steroid PRO was ‘relevant or very relevant’ to them and their disease. Feasibility: 97% (30/31) patients and 100% (14/14) HCPs reported the Steroid PRO was ‘easy or very easy to complete’. Patients reported that the four domains of the Steroid PRO had relevance to them and that it was validating to see their concerns represented: ‘It’s obvious you guys know what you’re talking about—these are my issues. It’s very validating when you realise it’s not just you. These problems are real and they matter.… These are not questions my doctor asks me about. Doctors never ask about psychosocial aspects. It would be really great if they used this’ (female patient with asthma). Patients and clinicians felt the Steroid PRO would be suitable for use in clinical practice within their specialties and would aid in understanding of the impact of glucocorticoids.

**Conclusions:**

The Steroid PRO demonstrated face validity and content validity for assessing the impact of glucocorticoids in patients with inflammatory respiratory, gastroenterological and dermatological conditions. Additionally, the feasibility of using the Steroid PRO with both patients and HCPs has been established. Future work should include quantitative testing of the Steroid PRO as an outcome measure within clinical trials in these conditions.

**Trial registration number:**

NCT06314451.

**STRENGTHS AND LIMITATIONS OF THIS STUDY**

- This is an international study of patients from the UK and USA with a range of different demographics and inflammatory respiratory, gastroenterological and dermatological conditions treated with oral glucocorticoids.
- Clinicians working in respiratory, gastroenterology and dermatology in the UK and USA were also included in the study.
- Cognitive interviewing methods were adapted for this cross-condition validation of Steroid PRO in different diseases to determine face and content validity and feasibility to patients and clinicians.
- Limitations of this study include focus on patients and clinicians from the UK and USA: further work will be needed to explore different countries and languages.
- Future work will also be needed to perform longitudinal quantitative testing of the Steroid PRO as an outcome measure in inflammatory respiratory, gastroenterological and dermatological diseases.

## Introduction

 Glucocorticoids (GCs) are essential treatments used within all medical specialties to control life-threatening and organ-threatening disease and manage symptoms.^1^,^8^ Despite the effectiveness of GCs, patients and clinicians are concerned about their wider impact^9^,^14^; adverse effects include mood, sleep and weight disturbance, and increased risk of infection, diabetes, hypertension and osteoporosis.^15^,^19^

A systematic review of quantitative and qualitative studies by the Outcome Measurement in Rheumatology Working Group explored the impact of GCs on patients, including asthma and obstructive airway disease, inflammatory bowel disease, immune thrombocytopenia purpura, systemic lupus erythematosus, antineutrophil cytoplasmic antibody (ANCA)-associated vasculitis and rheumatoid arthritis, adrenal insufficiency and multiple sclerosis.^20^ Physical symptoms (eg, weight gain, change in appearance and impact on sleep), psychological symptoms (eg, change in mood, anxiety and depression and personality change), effect on participation in daily life (eg, impact on family life and relationships, work and friendships) and contextual factors (eg, lack of support from family and friends, media and society due to negative connotations of taking GCs) were identified as of key importance to patients across these diseases.^20^ The need for a patient-reported outcome measure (PROM) to determine the impact of GC therapy on health-related quality of life (HRQoL) in adults with inflammatory diseases was highlighted.^21^

The US Food and Drug Administration defines a PROM as ‘any report of the status of a patient’s health condition that comes directly from the patient’.^22^ The Steroid PRO is a new 15-item scale, validated for measuring the impact of GC therapy in people with rheumatic diseases.^23^ Its validation study involved >900 patients with inflammatory rheumatic conditions from the UK, USA and Australia and has four subscales (Social impact, Impact on appearance, Psychological impact and Treatment concerns).^23 24^ The Steroid PRO can be used in clinical practice to support shared decision-making, by highlighting treatment impact from GCs from the patient perspective, and as a validated outcome measure in clinical trials.^23^

Given the similarities of GC impact in other inflammatory conditions, it seemed plausible that the Steroid PRO could be acceptable and effective for use with people with inflammatory lung, bowel disease and skin conditions, treated with GCs. The objective of this study is to examine face and content validity and feasibility of the Steroid PRO to patients and clinicians within respiratory/pulmonology, dermatology and gastroenterology departments in the UK and USA.

## Methods

This is a qualitative study using semistructured cognitive interview methods with patients with inflammatory conditions from respiratory/pulmonology, gastroenterology and dermatology specialties and healthcare professionals who care for them, from the UK and USA.

### Patient and public involvement

A steering committee of researchers, methodologists, clinicians and patient research partners oversaw the study, including study design, production and testing of informational material and data collection forms and interpretation of analysis. In the original Steroid PRO development and validation project, the same patient research partners contributed to final choice of items and nomenclature of domains^24^; and reported the final Steroid PRO questionnaire (1 page, 15 items) as an acceptable burden for participants. At the end of this study, patient research partners commented on the findings and contributed to the dissemination plan.

### Sampling and eligibility

Inclusion criteria for patients included diagnosed with a respiratory, gastroenterological or dermatological autoimmune or inflammatory condition, treatment with oral or intravenous GCs within the previous year (or longer), ability to give informed consent and 18 years or older.

Inclusion criteria for healthcare professionals included working within respiratory/pulmonology, dermatology or gastroenterology departments in the UK or USA.

### Recruitment

All participants were identified by their clinician in the UK and USA. For sites in the USA, a data transfer agreement was in place between the study PI institute and two universities in the USA. Potential participants from the UK and USA gave verbal consent for their contact details to be securely transferred to the research team to receive further information about the study. All participants then completed written informed consent prior to their inclusion in the study.

Potential healthcare professional participants were identified and approached within the same hospitals and through professional networks, with either initial contact from the research team or through clinical colleagues. Recruitment of patients and healthcare professional participants was guided by use of a purposive sampling framework to ensure a range of participants with different demographics and disease characteristics (patients) and role (healthcare professionals).

### Interview procedures

Interviews with patient and clinician participants were conducted face to face, via telephone or online, depending on participant preferences. Three researchers conducted the interviews (SB, A-MTS and JCR). A-MTS is a researcher and rheumatology nurse specialist, JCR is a researcher and rheumatology consultant and SB is a non-clinical researcher with prior experience contributing to the original development of the Steroid PRO. The research team reflected on and discussed how their professional backgrounds and experience might influence their interviewing techniques and analysis, particularly their prior work with individuals with rheumatic diseases receiving GCs and understanding the impact of these medications.

The Steroid PRO questionnaire^23^ was shared with participants during the interview, to capture participants’ initial reactions and responses to the questionnaire. The Steroid PRO is a 15-item questionnaire with 4 subscales: Social impact (4 items); Impact on appearance (3 items); Psychological impact (5 items); Treatment concerns (3 items).^23^ See online supplemental figure S1 for summary of the Steroid PRO subscales and items.^23^ Patient research partners report that the Steroid PRO takes less than 2 min to complete.

A data collection tool was designed to assess face and content validity and feasibility of the Steroid PRO in new populations. Analogous studies of concepts of applicability^25^ and sensibility^26^ when choosing measurement tools informed the development of the data collection tool. Patients and clinicians were asked “How relevant is the questionnaire to you and your disease/speciality?”—not relevant, relevant or very relevant and also “How feasible (easy to complete) do you think the questionnaire would be to complete at or before a clinic visit?”—not easy, reasonably easy, very easy?

The cognitive interviewing (CI) technique, used within the initial development stages of PROMs^24^ and also recommended for use in PRO Translatability Assessments,^27^ was adapted in this study to test the Steroid PRO in new disease-representing populations. CI was carried out using pragmatic and ‘light-touch’ interviewing, underpinned by think-aloud techniques and clean language principles.^28^ Participants were asked to give feedback on the content and structure of the Steroid PRO including overall impression of the questionnaire (acceptability, wording of stems and response categories and instruction for completion), and give short-answer responses to each of the 15 individual items in terms of understanding and importance to them. Demographics and disease features (patients) or role (healthcare professional) were recorded at the end of the interview.

### Analysis

Interviews were audio recorded, anonymised and labelled with a unique project ID. Audio recordings were checked and data transcribed. Data were managed via charting of participant responses in tables in Microsoft Word and Microsoft Excel software.

Responses to the relevance and feasibility (3-point Likert scale) questions were reported for patients and healthcare professionals separately by summarising results and percentages within Excel.

The short answer questions given within the CI stage of the interviews were first read and analysed for patients and healthcare professionals separately. A combination of deductive analysis (in relation to questions specifically about relevance and feasibility and individual item topics) and inductive analysis (used to identify new themes within the responses to open questions about the overall impressions of the questionnaire) were used.^29^ These longlists of themes identified by patients and healthcare professionals were then reviewed in parallel to identify commonalities and differences between them and their responses to the Steroid PRO. Patient participant responses to the individual items were examined within the four domains of the Steroid PRO (Social Impact; Impact on Appearance; Psychological Impact; Treatment Concerns). Convergent or divergent quotes to the items and domains were reported across and between patients treated for the three types of inflammatory disease, to describe the impact of steroids on HRQoL in these groups and to identify potential differences between them.

Demographic patient and clinician data were analysed using Excel software for Windows using mean and proportions as appropriate.

## Results

Patient and clinician participants were recruited from University Hospitals Bristol and Weston NHS Foundation Trust, Bristol, UK, University of Pittsburgh Medical Centre, Pittsburgh, USA and Johns Hopkins Medical Centre, Baltimore, USA and Mayo Clinic, Minnesota, USA.

A total of 42 patients were recruited from respiratory/pulmonology (n=14, 33.3%), dermatology (n=13, 31.0%) and gastroenterology (n=15, 35.8%) medical departments; 32 in the UK and 10 from the USA. Mean age was 48.2 years (range 22–71) and 19 (45.2%) were female. Patient participants reported a range of inflammatory respiratory, dermatological and gastroenterological conditions; demographics and patterns of GC use are reported in table 1 and online supplemental table 1.

**Table 1:** Patient demographics, inflammatory conditions and glucocorticoid (GC) use (n=42)

		Resp	Derm	Gastro	Total (%)
Age (years)	18–30	0	2	4	6 (14.3)
	31–65	13	8	9	30 (71.4)
	>65	2	2	2	6 (14.3)
Sex	Female	8	7	4	19 (45.2)
	Male	8	5	11	24 (57.1)
Resp conditions	Asthma	10			10 (23.8)
n=14 (33.3%)	Organising pneumonia	1			1 (2.4)
	Cystic fibrosis	1			1 (2.4)
	Interstitial lung disease	2			2 (4.8)
Derm conditions	Bullous pemphigoid		1		1 (2.4)
n=13 (31.0%)	Cutaneous systemic lupus erythematosus		1		1 (2.4)
	Cutaneous polyarteritis nodosa		1		1 (2.4)
	Dermatomyositis		1		1 (2.4)
	Eczema		4		4 (9.5)
	Exfoliative erythroderma		1		1 (2.4)
	Hidradenitis suppurativa		2		2 (4.8)
	Pemphigus		1		1 (2.4)
	Pyoderma gangrenosum		1		1 (2.4)
Gastro conditions	Crohn’s disease			7	7 (16.7)
n=15 (35.8%)	Liver transplant			1	1 (2.4)
	Ulcerative colitis			7	7 (16.7)
Current GCs (mg)	<7.5	6	9	10	25 (59.5)
	>7.5 and <30	7	2	2	11 (26.2)
	>30	2	1	3	6 (14.3)
Ethnicity	African American		1		1 (2.4)
	Black British			1	1 (2.4)
	Chinese		2		2 (4.8)
	Indian British	1			3 (7.1)
	Japanese			1	1 (2.4)
	Mixed White British/Black African		1		1 (2.4)
	Mixed White British/Arab	1			1 (2.4)
	South Asian			1	1 (2.4)
	White American	4	1	4	9 (21.4)
	White British	7	6	8	21 (50)
	White Polish		1		1 (2.4)

14 healthcare professionals participated in the clinician interviews: 9 from the UK and 5 from the USA. 11 were consultants/professors, 1 registrar and 2 specialist nurses. Clinicians worked in the three specialties (seven in respiratory/ pulmonology, four in dermatology and three in gastroenterology); nine were female and five were male. Nine healthcare professionals had over 10 years of experience in the specialty, 4 had between 5 and 10, and 1 had less than 5 years.

### Face and content validity and feasibility of the Steroid PRO

**Figure 1:**
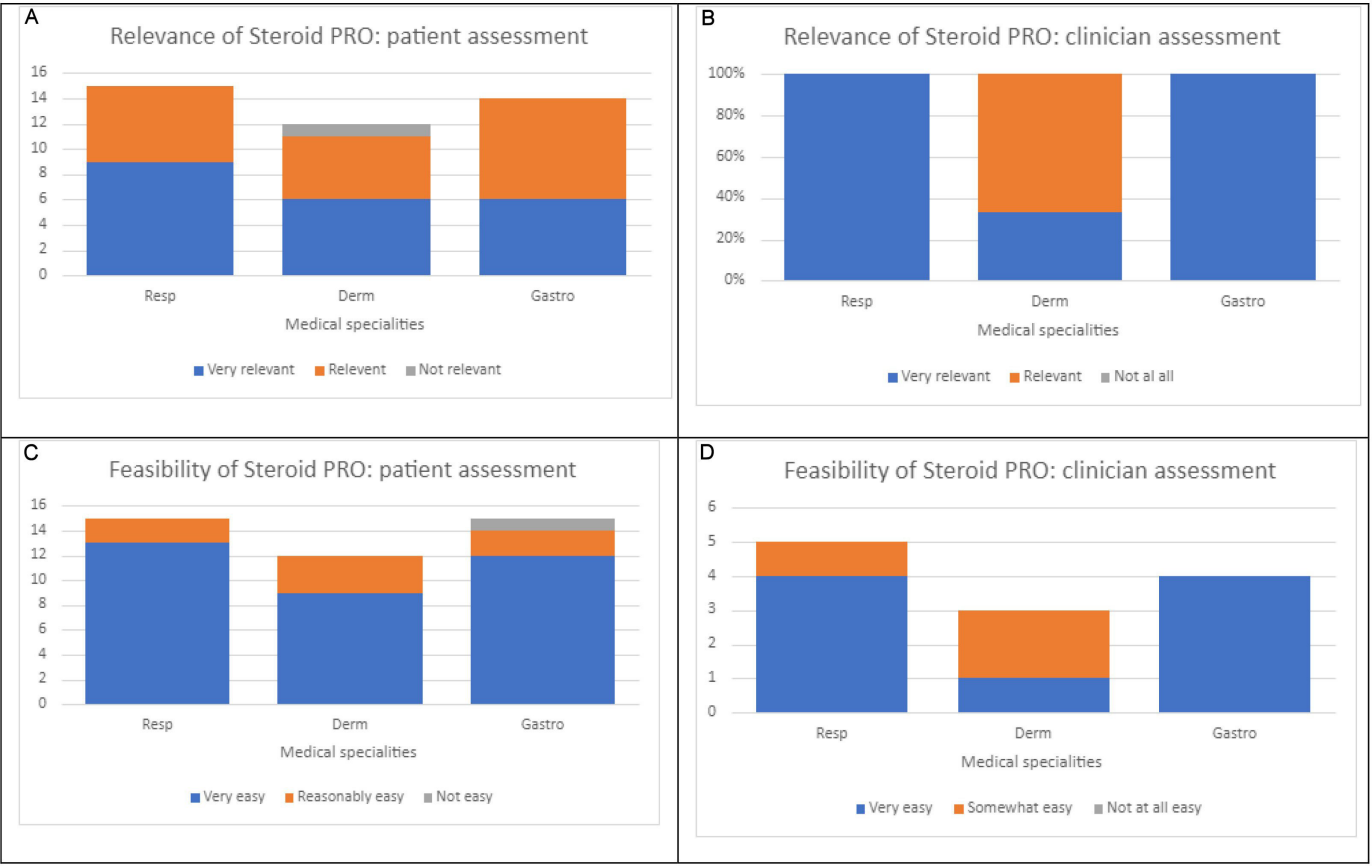
Assessment of the Steroid PRO by patients and clinicians in respiratory, dermatology, gastroenterology. (A) Patient assessment of relevance, (B) clinician assessment of relevance, (C) patient assessment of feasibility, (D) clinician assessment of feasibility.

In response to how relevant the questionnaire was to them, 41 of 42 patients reported that the Steroid PRO was either ‘very relevant’ or ‘relevant/somewhat relevant’, in terms of the impact of treatment with GCs for their inflammatory disease. The one patient who stated that the Steroid PRO was not relevant to them, reported this was because, ‘steroids were only given as short courses for my Eczema’ (F, Eczema) (see figure 1).

Almost all (n=41, 97.6%) patients reported that the Steroid PRO was either ‘very easy’ or ‘somewhat easy’ to complete. The patient who reported that the Steroid PRO was ‘not easy’ to use, said that they would ‘prefer to talk about their symptoms rather than tick boxes’ (M, Crohn’s Disease).

All 14 clinicians reported that the Steroid PRO questionnaire was relevant to their patients and their treatment with GCs, and either very easy or somewhat easy to complete for patient participants in their clinics to complete (see figure 1).

### Face and content validity: short answer responses analysis

#### Focus on the patient perspective

Patients reported that they liked how the Steroid PRO focused on how they felt and measured the patient perspective of the impact of steroids:

It’s obvious you guys know what you’re talking about—these are my issues. It’s very validating when you realise it’s not just you. These problems are real and they matter.… feel I’m just complaining. These are not questions my doctor asks me about. Doctors never ask about psychosocial aspects. It would be really great if they used this. I wish they used this. (F Asthma).

The questions kind of cover everything, like physical and mental. It’s about how you’re feeling in yourself as well, which is good (F Crohn’s Disease).

Clinicians were also positive above how the Steroid PRO focused on the patients’ perspective of the impact of steroids and gave people permission to raise potentially sensitive issues within their consultations:

I think the following [introduction] sentence is quite helpful—there is no right or wrong answers and it’s to do with their experience of steroids. (F Dermatology Consultant).

Easy for the patients to fill in and particularly picking up on things that they would less freely volunteer—it does it in a way that validates, you know ’you’re not the only one that might experience that’—I think it gives them the ability to put that down in a way that’s not embarrassing. (F Respiratory Consultant).

#### Relevance to people with respiratory/pulmonology, dermatological and gastroenterological conditions in clinical practice

Patients reported that the Steroid PRO would be relevant to their disease and its treatment:

I think it’s very important because if it does affect people, they’re letting you know that it’s affecting them. Definitely relevant [to respiratory]. (F Asthma.)

There’s a lot of things in there that relates to me and how I felt, so I’d say there’s only a few that didn’t affect me. So I think it is quite relevant to me. (F Pemphigus)

I think it’s very good- it’s relevant across the board. Lots of people taking it for different conditions. I think it’s relevant for just about everybody (M Crohn’s disease).

Clinicians also reported that the Steroid PRO would be relevant to their patients and practice:

Yeah, I think it is relevant. We’re all very aware these days of the issues we’re causing by giving people steroids so I think this is a very good way of capturing that data. If you can compare data over a period of time with people who are on steroids for a long period of time, you’ll see some these things getting more relevant to that person. You’ll see those changes. (F Respiratory specialist nurse).

I think if we use, if you use this to educate the providers or the physicians or the caretakers, and I think it is very important that we learn this from the patients (M Pulmonology Consultant).

#### Nomenclature of GCs

The nomenclature related to GCs was raised as an issue. In the original Steroid PRO questionnaire, the terms ‘steroids’ and ‘prednisolone’ are used; patients and clinicians highlighted other types of oral steroids:

In the IBD [inflammatory bowel disease] population, there are lots of people on other steroids [than pred], so I think I might feel like this wasn’t designed for me. The rest of it seems very clear. (M Ulcerative colitis).

There are a wide range of steroids…some people don’t realise what they’re on, if they’re on something slightly unusual…you can’t really put a list of all of them, so I think that’s fine! We’d tend to use similar, but in gastro they’d use budesonide…none of it sounds like prednisolone. (Dermatology Consultant).

#### Attribution of adverse effects

Attribution was raised as a difficulty with some participants in terms of determining between impact of the treatment and the disease itself:

Some of the experiences are related to the disease, so I get quite tired when I have a flare up, but the steroids then boost energy so it’s a bit difficult trying to distinguish between the condition and the effect of the drugs. I think the question hit the relevant points. (M Ulcerative Colitis).

### Feasibility of the Steroid PRO: analysis of short answer responses

#### Design of the Steroid PRO

Patients reported that the design of the PRO made it easy to use. The length, wording of items and response options were found to be acceptable, and the tick box response options made it easy to complete.

Yeah, I think that’s very reasonable. Personally I wouldn’t have any issue filling out a longer questionnaire. (F Eczema).

It’s nice to have something where you can literally tick a box. (M 58, Cutaneous Polyarteritis Nodosa, UK).

I wouldn’t have a problem filling it out, I have had a fair few side effects in all fairness. For me, being dyslexic, there’s nothing [problematic], I can read it all and understand it all. (M Pyoderma Gangrenosum).

In terms of feasibility, clinicians felt the Steroid PRO could be incorporated into clinic settings.

They’re quite easy, not difficult questions, and a nice tick box, no asking them to write anything so that’s good. When I look at some of the questionnaires we give to people, that is nothing compared to them. (F Respiratory specialist nurse).

I think it is very easy. I think it takes patients at the most—10 minutes—even if they think about every question. (M Pulmonologist)

Yeah, quite helpful in terms of the side effects that people get, they might barely get something, but it’s something that’s impactful for them…it would capture all of those. It gives you a reasonable time period and I think having a wide spectrum in terms of how problematic it is for them, it’s easy for patients to answer that I think. (F Respiratory Consultant).

### Patient feedback on individual items and domains of the Steroid PRO between conditions

Short-answer responses to each of the individual items within the four domains of the Steroid PRO (Psychological Impact, Impact on Appearance, Social Impact and Treatment Concerns) were charted and reviewed across conditions to identify commonalities and differences between groups (dermatology, gastroenterology and respiratory/pulmonology).

#### Psychological impact

The psychological impact of steroid treatment was significant to patient participants across conditions, including change in mood, restlessness and feeling agitated (see box 1).

Box 1 **Psychological impact domain, quotes from patients about the impact of glucocorticoids**

**Psychological impact**

**Q1 Physical agitation**

*It’s relevant. Sometimes I’m restless but I don’t think I’m physically agitated. [pause, possibly listening to husband in the background] …my husband thinks I am physically agitated. Because I’ve been on steroids so long, I think it’s very relevant to me*. (F Exfoliative Erythroderma)

*I recognise it—if I’m taking oral steroids rather than inhaled steroids that would be very much the case. I recognise the agitation*. (F Asthma)

**Q3 Anxiety**

*I am not an anxious person by nature, but I would get anxious, like panic attacks* (F Bullous pemphigoid).

*Anxiety, I get. I think, ’what is it going to do this time… am I going to get jittery and horrible again?’ —I don’t know if I overthink when I know I’m having to take a higher dose. The anxiety then makes you feel a bit low because of it*. (F Asthma)

**Q4 Clarity of thinking**

*In terms of making decisions… my usual way of making decisions is very based off of numbers, facts, that sort of thing… with the decisions I was making during that time, my ability to make decisions—it was very much, ’let’s just do it’… taking less information and making a very bold—kind of statement—decision. I think it was a significant difference in the way that I made the decision*. (M Ulcerative Colitis)

*When it’s really bad it can affect me tremendously, and make some very unwise decisions. If I’ve been on a short course, it’s not as bad, but when I’ve been on a long course these things happen the most*. (F Asthma)

**Q5 Talking too much**

*When I’m chatting to neighbours I feel like I’m waffling on, and they’re like, ’[name], I’ve gotta go’*. (M Pyoderma Gangrenosum)

*Yeah, when I’m on steroids that’s right, that does happen. They make you a bit more braver or bolder I think. Probably too fast, I would say that was steroid-induced. I didn’t care about it so not that important to me! So woe betide anyone around me!* (F 49, Dermatomyositis, UK)

**Q8 Anger/irritation**

*Yeah, I did feel irritable. Maybe like a slightly shorter fuse or something. I think people around me, probably the people that are closest, noticed*. (M 42, Ulcerative Colitis, UK)

*Most definitely! I certainly warned my family and friends…actually having to explain to my kids what ’road rage’ was!—just educating those around me. I definitely recognise now when it’s to do with normal life or whether it’s steroid-induced*. (F Dermatomyositis)

Yes, I was on quite a high maintenance dose, and most definitely felt restless and agitated! It was like being on speed! (F Dermatomyositis).

Yeah, I have felt confused. I’d think, ‘I’ve got to turn the heating on or off’, and I’d go upstairs and think, ‘oh, I’ve got to do that as well’, and I’d come back down and like, I’ve gone in the bathroom and forgotten to do both of the things like. (M Pyoderma Gangrenosum).

I was just exhausted. Constant exhaustion… I think you can get brain-exhausted when you think a lot, or you can get physically exhausted if you’ve done a lot, but this was just exhausted with living—I just ran out of steam. When I was on the medication it just kind of wiped me out. (F Crohn’s disease)

#### Impact on appearance

This domain includes items related to weight, change in appearance and impact on clothes and had a high saliency with almost all patients regardless of condition (see box 2).

Box 2 **Impact on appearance domain, illustrative quotes from patients about glucocorticoids**

**Impact on appearance**

**Q2 Appearance**

*I work out in the public, that is something and being a larger woman pretty much all my life, I’ve lost a good bit of weight. When we had to really ramp up the steroids so I could breathe, with the wheezing so bad—and it’s painful to wheeze. It was very disheartening to gain back of like 50 pounds. But I had been really trying to get off this, so that moonface, it’s frustrating sometimes* (F Asthma).

*Yeah, it made me put on weight, which I didn’t like at all. It is my vanity speaking [laughs] but yeah, for me it was very important* (M Ulcerative Colitis)

*My face has definitely got more puffy. My friends have said that they notice it too. My skin’s got worse—drier and a bit more spotty than normal. I don’t think I’m necessarily unhappy with it because I know it will go back to normal. It does affect my self-confidence*. (F Crohn’s Disease)

*Yeah, one of my main things. I put on a lot of weight, it happened quite fast. I have struggled with that. I was very puffed up in my face. It isn’t a time I don’t like to look back on. It has really affected me*. (F Crohn’s Disease)

**Q12 Weight**

*I honestly think that’s the main thing with this prednisolone is the weight gain. That’s the problem that a lot of people struggle with because it’s such a drastic change so fast when you take it. I have to take it every day and my appetite is nonstop, even when I don’t wanna eat, I’m still hungry* (M Hidradenitis Supporitiva)

*Wooah—yeah! It’s steroids, yes! Definitely. When you’re gaining weight from the steroids, it starts affecting your joints also* (F Cutaneous Systemic Lupus Erythematosus)

*Yes—it was a trouble, I had a huge appetite, I couldn’t stop eating. I think I put lots of weight on as well as puff up [steroid side effect] so that was a big trouble, yes*. (M Ulcerative Colitis)

*It made me really hungry…I could just tuck away food. I didn’t realise that I was about to stack it on really quickly. And suddenly, I didn’t fit my jeans at all. the weight just suddenly appeared. Second time on steroids, I was a lot more mindful about eating, I knew the effects. I was a lot more controlled. But I still put on weight really quickly—I was so reluctant to go on them knowing that I put on over ten kilos in the first round*. (F Crohn’s Disease)

**Q14 Clothes**

*Lord I deal with this! Yes, this is big! I’ve had to go shopping recently because sweatpants or shorts or T-shirts don’t fit. I went from wearing an XL—now I’ve been 2XLs* (M Hidradenitis Suppurativa)

*Oh yeah, definitely. [People on steroids] have two different [wardrobes], one when we are on high doses, and when it’s better we have different clothes*. (F Cutaneous SLE)

*Definitely. The amounts of money I’ve had to spend buying new clothes—I’ve got clothes from a size 6 to an 18. Opening the wardrobe and having that constant reminder… all these lovely clothes—I haven’t got the confidence anymore, I won’t wear that, can’t wear that—I’m very limited on what I can wear—and that was hard. It’s horrible*. (F Asthma)

It’s very very relevant. My appearance has changed hugely since I’ve been on long-term steroids—yes, my face, the shape of my eyes physically. (F Exfoliative Erythroderma).

A lot of it is muscle wasting. A lot of people experience chipmunk cheeks. Mine is more muscle wasting, and a spare tyre around my middle. (M Crohn’s Disease).

Yeah—It’s really knocked my confidence. I don’t feel comfortable in my own skin anymore. I’ve had people say ’you can tell you’re on steroids because you’ve got moon face’… it makes you feel awful. (F Asthma)

However, the issue could be nuanced, for example, a patient with inflammatory bowel disease reported:

Because my weight was dropping from my illness, the steroids actually bringing my appetite back was more of a positive thing (M Ulcerative Colitis)

One dermatology patient reported that steroids overall had a positive impact on appearance:

In terms of topical steroids, that might be a little more relevant, but oral steroids, once you get to that point where you need oral steroids, you’re kind of like, I don’t care what they do to me—as long as it clears up my eczema, I’m happy (F Eczema).

#### Social impact

Patients from all condition groups reported impact on joining in and social interaction due to their steroids, due to changes in mood, energy and appearance (box 3).

Box 3 **Social impact domain, illustrative patient quotes from patients taking glucocorticoids**

**Social impact**

**Q7 Fatigue/tiredness**

*I’ve suffered terribly with insomnia since I’ve been on steroids, it’s a very very nasty circle. As soon as I sit down in a chair to relax I fall asleep because I’m so tired, but I’m not sleeping at night. But I would say the problem with fatigue and tiredness is due to the insomnia. It’s a bit of a vicious circle*. (F Exfoliative Erythroderma)

*This is me all the time. Insomnia because of the steroids. You don’t sleep at night and during the day you are exhausted*. (F Asthma).

**Q10 Being with other people**

*Every time, because (a) I didn’t want to get irritable with them, and (b) I didn’t feel comfortable in my own skin. I don’t feel that I’m very good company when I’m on steroids as well—I feel a lot lower, almost like depression. The last thing I wanna do is go out and get dressed up, look for an outfit, that I’m not comfortable in*. (F Asthma)

*I don’t think I avoided being with other people but I just got ’peopled out’ really quickly, if that makes sense. I just don’t have capacity to be around people, I’m just exhausted. I wasn’t even, like, messaging—I just didn’t feel right in myself, I didn’t want to talk to people. I was just fighting to get through each day really*. (F Crohn’s Disease)

*Yes, I think I do suffer with being sometimes quite irritable. My husband has said that I snap at him, when I don’t realise that I am*. (F Exfoliative Erythroderma).

**Q13 Joining in**

*When I’m out and meeting new people… I avoid joining in. It’s had a massive effect. Because I’m sleeping half as much I’m not able to focus as well, so joining in conversations I miss half the conversation… people think I’m being rude and I’m not interested… when it couldn’t be further from the truth*. (F Asthma)

*Yeah. After I finished work, I felt too tired to engage with my children, I just wanted to have a nap, so the low energy side of that has certainly been impactful*. (M Ulcerative Colitis)

**Q15 Everyday responsibilities**

*Because of my lack of energy [on high steroid dose] I couldn’t really do things around the house. I wanted to. I couldn’t do anything, I was just so tired. I can do them now*. (F Pemphigus)

*I’m retired, I look after my grandchildren, so in terms of child care, yeah, I don’t have the energy, and I’m irritable, so it’s both*. (F Asthma)

*Yeah, I feel like I’ve let [people] down, and with supporting friends and family*. (F Asthma)

*The steroids made me wake up really early, no matter how much sleep I’d had or how late. I work shifts and I’d still be waking up normally about 4.30 or 5 in the morning. I was wide awake. So at work I’m then…exhausted. I was sleeping maybe three to 4 hours a night tops, every night, and I knew that was affecting my performance at work. It really put me in a low mood. It was brutal*. (F Crohn’s disease)

*Yeah, definitely, I’d say always. Definitely had problems with it …with the kids, it puts a lot of pressure on my husband, and I feel I can’t be there for them* (F Eczema)

It’s a weird one—they make me wired, but they also make me a bit depressed—so I think it’s not that I’d say I’m not a nice person to be around… but I just don’t feel like seeing other people. I literally haven’t got the energy to be able to chat to people… and I’m wired at the same time, it’s horrible. (F Asthma)

#### Treatment concerns

Patients expressed concerns and reported feeling annoyed and upset about GC treatment, with regard to adverse effects, short-term and long-term risks and lack of alternatives (box 4).

Box 4 **Treatment concerns domains, illustrative quotes from patients about glucocorticoids**

**Treatment concerns**

**Q6 Feeling upset (about taking steroids)**

*Yes I would say very much so. Having to remember to take them, and then being conscious that there were some side effects and I guess to some extent looking out for them*. (F Ulcerative Colitis)

*That’s very important to me. Yes, I do feel upset, and I feel annoyed about the need to take steroids cos it’s completely changed my life*. (F Exfoliative Erythroderma)

*Yes! I felt upset and annoyed about having to take steroids I must say. I hate taking any medication and… I felt, I shouldn’t be on these. I’ve been fit all my life and now this has happened. It was the last thing I wanted to be on*. (M Crohn’s Disease)

**Q9 Extra medications**

*I do. Sometimes I think, “I’m on so many tablets, my god.” D’you know what I mean? I wish I didn’t have to take them. I just wish I was OK. When I go away with the girls for a weekend they say “oh my god how many tablets are you on?", and I think, I am on a lot of tablets—it’s worrying. I do think about it. [Question is] clear, brilliant examples. You’ve got to take them [extra medications], to take that [risk of steroid-induced disease] away*. (F Asthma)

*I’m on so many different medications. I wonder if the side effects are causing the problems I’m having* (M Organising Pneumonia).

*It’s more annoying than upsetting. I take 33 tablets a day, I come down [in the morning] and I’ve got to get 19 tablets out*. (M Pyoderma Gangrenosum).

*It’s more the cost of it really. Arguably it is [having an effect on my life], especially with the financial climate at the moment, every penny counts. It’s nearly £10 per prescription now*. (M Ulcerative Colitis)

**Q11 Long-term risks**

*Yeah, I have worried about the long-term effects, clearly. I mean, I have got really high blood pressure as well as osteoporosis*. (F Asthma)

*Yes, very much so. What it’s doing to my body, and I’m aware that you can’t be on steroids for a very long period. Having to come on them and off them and on them and off them—it’s that worry of what that’s doing to my body long term*. (F Ulcerative Colitis)

*I do think about it. I’ve been taking them for 13 years. I know you shouldn’t be taking them for that long. You’re only supposed to be on ’em for a short time. To be honest I don’t think it has affected me at all apart from just thinking about it. It does cause a bit of a worry*. (M Asthma)

*Yes, definitely. It did concern me to know that every day I was being put on more steroids—that definitely racked my brain a little bit*. (M Ulcerative Colitis)

I did feel this—I spent the last so many months saying I really wanna get off steroids—I really didn’t want to take steroids and I did feel upset. (F Pemphigus).

Yeah, 100%, always. Surely in this day and age there should be something else that can help? Something that’s not got all these side effects. It upsets me and frustrates me a lot. (F Asthma)

Very upset. It was catch-22; I was happy with how they were keeping my symptoms in check but unhappy and anxious about needing to take them and worried- will I ever be able to stop taking them? (M Ulcerative colitis).

One patient taking Clipper, which is a type of GC designed to take effect within the bowel only, rather than being systemically active, reported:

I don’t think I did feel annoyed about having to take them. More recently I’ve been on Clipper, which I’ve felt I’ve had far less side effects from the prednisolone. (M Ulcerative colitis)

## Discussion

The Steroid PRO is a patient-reported outcome used to capture the impact of GCs from the patients’ perspective, originally developed and validated in the rheumatic diseases.^23^ This study has demonstrated face and content validity and feasibility for use by patients with inflammatory respiratory/pulmonology, dermatological and gastroenterological conditions. The items and domains within the Steroid PRO (Social Impact, Impact on Appearance, Psychological Impact and Treatment Concerns) are relevant to patients and healthcare professionals working within these specialties. The Steroid PRO appears to be acceptable for use in clinical practice as a communication tool between patients and their clinicians and could capture the patient perspective of GCs within clinical trials.

Patients and healthcare professionals valued the emphasis on the psychological and emotional impact of GC treatment within the Steroid PRO and felt this tool could act as a prompt to clinicians to ask more about these aspects. These discussions could also validate peoples’ experiences (eg, change in mood, energy and appearance), which are common upsetting side effects that are not usually discussed in clinic appointments. In this way, the Steroid PRO could help shared decision-making between patients and their clinicians, for example, when designing steroid reduction plans and alternative immunosuppressant therapies.

The strength of this study is that patient participants were recruited from a range of different medical specialties, with different conditions and demographics, from the UK and USA. A spectrum of health professionals working across specialties was also included.

This is the first assessment of the Steroid PRO in these populations, and larger-scale quantitative evaluations will be needed to test its use further. Other limitations of PROs in general were identified by participants, for example, the difficulty of ascertaining cause of certain symptoms, for example, fatigue and tiredness can be due to disease or GCs. This is a common limitation of PROs and may be addressed within further validation studies looking at correlations with other measures which may explore causation in more depth.

Interestingly, the Steroid PRO feedback did differ in some regard based on condition, for example, in dermatology patients, where disease symptoms can alter facial appearance, the impact of steroids on the appearance change domain may be more complex. Within gastroenterology, patients can suffer significant weight loss during acute phases of their disease, so the weight gain that is seen with steroids (a negative for most patients), is seen as a positive for those with inflammatory bowel disease. Also, note was made that patients taking steroids whose mode of action is within the bowel wall (such as Clipper), reported fewer adverse effects. People with respiratory disease reported the most significant impacts were seen when taking oral or intravenous/systemic GCs rather than inhaled GCs. People taking inhaled GCs alone were not included in this sample, this is a potential limitation and could be explored more in future studies of the Steroid PRO. Both patients and health professionals highlighted that names could differ for certain GCs in common use within the different specialties, for example, budesonide in gastroenterology in the UK. These could be added to the introduction without changing the meaning or statistical validation of the Steroid PRO.

In relation to other qualitative studies on the impact of GCs in conditions such as asthma and inflammatory bowel disease, similar topics of importance, such as impact on weight, appearance, sleep, mood and participation, have been identified.^11 13^ The original systematic review across diseases also identified common themes to the Steroid PRO development.^20^ There is also a disease-specific GC impact PRO—the Systemic Lupus Erythematosus (SLE) Steroid Questionnaire^30^ which includes similar domains but is tailored specifically for patients with SLE.

In summary, this is the first study to confirm face and content validity and feasibility of the Steroid PRO for use in inflammatory respiratory/pulmonology, dermatological and gastroenterological conditions. The Steroid PRO could be useful in clinical practice to aid communication and shared decision-making, and as an outcome measure within trials to capture the impact of GCs from the patient perspective. Specifically, the Steroid PRO could be included as an outcome measure in trials of novel steroid-sparing biological agents—and potentially highlighting the benefit of reducing GCs use. The questionnaire items refer to the previous 7 days, making it adaptable for used as an outcome measure at any time point required during follow-up, regardless of study design. The validation study in rheumatology patients demonstrated that the Steroid PRO was psychometrically robust and suitable for use as a repeated measure in longitudinal studies and trials. Future validation work should include larger-scale quantitative validation of the Steroid PRO to test psychometric equivalence and responsiveness in different inflammatory conditions. Further work will also be needed to explore use of the Steroid PRO in different countries and languages. As a generic treatment-related PRO, the utility of the Steroid PRO could also be explored within other medical specialties, for example, oncology, haematology, neurology and infectious diseases to facilitate cross-condition comparisons in HRQoL due to treatment with GCs.

## supplementary material

10.1136/bmjopen-2024-089225 online supplemental file 1

10.1136/bmjopen-2024-089225 online supplemental file 2

## Data Availability

All data relevant to the study are included in the article or uploaded as supplementary information.
