# Supplementary material for: Impact of glucocorticoids on patients’ quality of life: a qualitative study assessing face validity and feasibility of the Steroid PRO in patients with inflammatory gastroenterology, respiratory and dermatology conditions
Source: BMJ Open. 2025 Feb 5;15(2):e089225. doi: 10.1136/bmjopen-2024-089225 (PMC11800201; doi:10.1136/bmjopen-2024-089225)
Supplement: online supplemental file 1 [file bmjopen-15-2-s001.pdf]

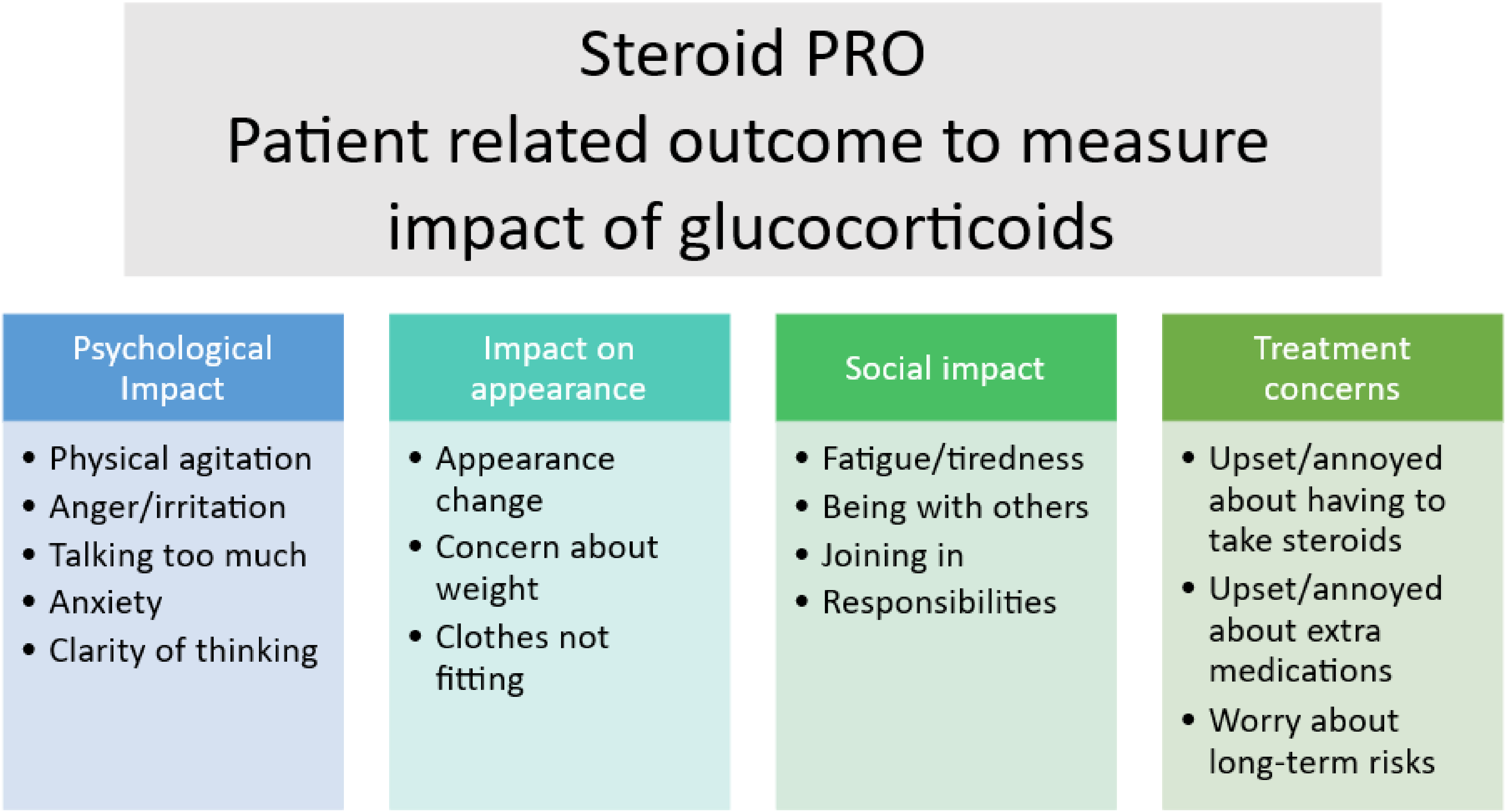
Steroid PRO
Patient related outcome to measure impact of glucocorticoids
Psychological Impact
Physical agitation
Anger/irritation
Talking too much
Anxiety
Clarity of thinking
Impact on appearance
Appearance change
Concern about weight
Clothes not fitting
Social impact
Fatigue/tiredness
Being with others
Joining in
Responsibilities
Treatment concerns
Upset/annoyed about having to take steroids
Upset/annoyed about extra medications
Worry about long-term risks

Figure 1
