# Supplementary material for: Impact of glucocorticoids on patients’ quality of life: a qualitative study assessing face validity and feasibility of the Steroid PRO in patients with inflammatory gastroenterology, respiratory and dermatology conditions
Source: BMJ Open. 2025 Feb 5;15(2):e089225. doi: 10.1136/bmjopen-2024-089225 (PMC11800201; doi:10.1136/bmjopen-2024-089225)
Supplement: online supplemental file 2 [file bmjopen-15-2-s002.docx]

**Table S1. Further demographic and glucocorticoid (GC) use patient interview participants (n=42)**

|  |  | **Resp** | **Derm** | **Gastro** | **Total (%)** |
| --- | --- | --- | --- | --- | --- |
| **First course of GCs** | <12 months ago | 2 | 2 | 2 | **6 (14.3)** |
|  | 1-5 years ago | 4 | 7 | 4 | **15 (35.7)** |
|  | 5-10 years ago | 2 |  | 3 | **5 (11.9)** |
|  | >10 years ago | 7 | 3 | 6 | **16 (38.1)** |
| **Longest GC course** | <6 weeks | 4 | 3 | 4 | **11 (26.2)** |
|  | 6 weeks- 6 months | 3 | 3 | 7 | **13 (31.0)** |
|  | 6 months- 2 years | 5 | 5 | 1 | **11 (26.2)** |
|  | 2-5 years | 1 |  |  | **1 (2.4)** |
|  | >5 years | 2 | 1 | 3 | **6 (14.3)** |
| **Education level** | No formal qualifications | 2 |  | 1 | **1 (2.4)** |
|  | School/high school | 4 | 6 | 3 | **13 (31.0)** |
|  | Degree/college | 8 | 6 | 8 | **22 (52.4)** |
|  | Vocational qualifications | 1 |  | 2 | **3 (7.1)** |
| **Employment** | Disabled | 2 | 2 |  | **4 (9.5)** |
|  | Employed | 5 | 5 | 8 | **18 (42.9)** |
|  | Retired | 3 | 1 | 2 | **6 (14.3)** |
|  | Student | 1 |  |  | **1 (2.4)** |
|  | Unemployed |  | 2 | 1 | **3 (7.1)** |
